# Ultrasound Features of Female Adnexal Tumor of Probable Wolffian Origin (FATWO): A Case Series and Literature Review

**DOI:** 10.2174/0115734056485227260724115008

**Published:** 2026-09-11

**Authors:** Li Xu, Lei Ye, Ketan Chu, Junmei Wang

**Affiliations:** 1 Department of Ultrasound, Women’s Hospital, School of Medicine, Zhejiang University, Hangzhou, Zhejiang, China; 2 Department of Pathology, Women’s Hospital, School of Medicine, Zhejiang University, Hangzhou, Zhejiang, China; 3 Department of Gynecology, Women’s Hospital, School of Medicine, Zhejiang University, Hangzhou, Zhejiang, China

**Keywords:** Female adnexal tumor of probable Wolffian origin (FATWO), Ultrasonography, Adnexal mass, Misdiagnosis, Immunohistochemistry, Rare gynecologic neoplasm, MRI

## Abstract

**Introduction:**

Female adnexal tumor of probable Wolffian origin (FATWO) is an extremely rare gynecologic neoplasm derived from mesonephric duct remnants. It exhibits nonspecific clinical and imaging features, frequently leading to preoperative misdiagnosis. To date, ultrasound findings of FATWO remain poorly characterized, with limited systematic reports. This study summarizes the ultrasonic manifestations of four pathologically confirmed FATWO cases and reviews the literature, aiming to improve preoperative recognition and fill the gap in systematic ultrasound descriptions of FATWO.

**Case Presentation:**

Four patients with pathologically proven FATWO were retrospectively analyzed. All tumors were located in the adnexal region and outside the ovary. Two cases presented as solid hypoechoic masses with clear boundaries and internal blood flow; one showed a cystic-solid mass with septations and mild vascularity; one appeared as a small cystic hypoechoic lesion. Preoperative ultrasound misdiagnoses included fibroma, corpus luteum cyst/endometrioma, granulosa cell tumor, and uterine leiomyoma. Pathologically, all tumors demonstrated typical tubular, cystic, and reticular structures. Immunohistochemistry was positive for CK, inhibin, WT-1, and calretinin, and negative for GATA-3 and PAX8. All patients remained disease-free during follow-up of 16–83 months.

**Conclusion:**

FATWO typically manifests as a well-circumscribed, extra-ovarian solid or cystic-solid adnexal mass with mild-to-moderate internal blood flow. When these ultrasound features are identified, FATWO should be included in the differential diagnosis to reduce preoperative misdiagnosis.

## 1 INTRODUCTION

Female adnexal tumor of probable Wolffian origin (FATWO) is an exceedingly rare gynecologic neoplasm with low malignant potential, which is derived from mesonephric (Wolffian) duct remnants [1]. Since its initial description in 1973, fewer than 100 cases have been reported globally. The majority of lesions occur in the broad ligament or mesosalpinx, and the clinical and imaging characteristics are non-specific [2].

Preoperative diagnosis remains extremely challenging, and the majority of cases are misdiagnosed as ovarian fibroma, granulosa cell tumor, cyst, or leiomyoma. Imaging investigations, particularly ultrasound, serve as the first-line examination for adnexal masses; however, the ultrasonic features of FATWO have not been well-defined. Published reports are limited, and most concentrate on pathology or magnetic resonance imaging (MRI) rather than systematic ultrasound findings [2-4].

Therefore, this study aimed to summarize the ultrasonic features of four pathologically confirmed FATWO cases from a single institution, compare them with previously reported cases, and identify key imaging indicators to enhance preoperative recognition. The study adheres to the CARE guidelines for case series reporting.

## 2 CASE PRESENTATION

This was a retrospective case series study approved by the Ethics Committee of Women’s Hospital, School of Medicine Zhejiang University, China. Written informed consent was obtained from all patients or their legal representatives prior to study inclusion.

### 2.1 Study Period and Case Selection

We reviewed all gynecologic tumor patients treated at our hospital between January 2018 and June 2024. Four patients with pathologically confirmed female adnexal tumor of probable Wolffian origin (FATWO) were enrolled. These were non-consecutive, clinically encountered cases collected over the study period due to the extreme rarity of the tumor.

### 2.2 Ultrasound Imaging

All patients underwent transvaginal pelvic ultrasound using:

(1) Samsung WS80A ultrasound system with an intracavitary probe

(2) GE E6/E8 ultrasound system with an intracavitary probe

Ultrasound examinations were performed and interpreted by two senior attending radiologists specializing in gynecologic imaging who were blinded to the final pathological diagnosis. Evaluated features included lesion location, size, echo pattern (solid/cystic/mixed), boundary, septa, internal vascularity, and resistive index (RI).

### 2.3 Pathologic and Immunohistochemical Analysis

Surgical specimens were fixed in 10% neutral-buffered formalin, processed routinely, and embedded in paraffin. Sections (4 μm) were stained with hematoxylin and eosin (HE) for morphological evaluation. Immunohistochemical staining was performed using the EnVision method for the following markers: CK, inhibin, WT-1, calretinin, GATA-3, and PAX8. All slides were reviewed by two senior gynecologic pathologists to confirm the diagnosis of FATWO.

### 2.4 Follow-up

Clinical follow-up was conducted *via* outpatient visits or telephone interviews. Follow-up data included survival status, tumor recurrence, and metastasis, with the last follow-up completed in October 2025.

### 2.5 Clinical Data

#### 2.5.1 Case 1

A 27-year-old female presented with a pelvic mass. Ultrasound showed a 2.7×2.0×2.5 cm hypoechoic mass adjacent to the right ovary (Fig. **1a**), with internal vascularity (Fig. **1b**), RI 0.72. It was misdiagnosed as fibroma. Pathology confirmed FATWO.

#### 2.5.2 Case 2

A 42-year-old female presented with a pelvic mass. Ultrasound showed a 2.3×2.3×1.7 cm cystic-solid mass in the left adnexa (Fig. **2a**–**c**), misdiagnosed as corpus luteum cyst or endometrioma. Pathology confirmed FATWO.

#### 2.5.3 Case 3

A 41-year-old female presented with a pelvic mass. Ultrasound showed a 5.1×3.9×3.2 cm septated cystic-solid mass (Fig. **3a** and **b**), misdiagnosed as granulosa cell tumor. Pathology confirmed FATWO.

#### 2.5.4 Case 4

A 53-year-old female presented with menstrual disorder. Ultrasound showed a 1.3×1.2 cm anechoic lesion adjacent to the left ovary (Fig. **4**), misdiagnosed as a simple ovarian cyst. Pathology confirmed FATWO.

### 2.6 Follow-up Results

Follow-up was completed in October 2025.

#### 2.6.1 Case 1

Follow-up duration was 83 months (January 2018 to October 2025). No recurrence, no metastasis, no additional treatment, no reoperation.

#### 2.6.2 Case 2

Follow-up duration was 75 months (July 2019 to October 2025). No recurrence, no metastasis, no additional treatment, no reoperation.

#### 2.6.3 Case 3

Follow-up duration was 32 months (February 2023 to October 2025). No recurrence, no metastasis, no additional treatment, no reoperation.

#### 2.6.4 Case 4

Follow-up duration was 16 months (June 2024 to October 2025). No recurrence, no metastasis, no additional treatment, no reoperation.

All patients were alive with no evidence of disease (NED) at the last follow-up. The clinical, ultrasonographic, and pathological features of the four patients are summarized in Table **1**.

### 2.7 Imaging Findings

#### 2.7.1 Case 1

Using a Samsung WS80A intracavitary probe for uterine and bilateral adnexal scanning, a 2.8×2.4×2.4 cm hypoechoic mass with well-defined borders (Fig. **1a**) and internal blood flow signals was observed adjacent to the right ovary, with an RI of 0.72 (Fig. **1b**). Pelvic magnetic resonance imaging (MRI) revealed a well-defined oval solid mass in the right adnexal region. On T1-weighted axial imaging, the mass appeared isointense to skeletal muscle (Fig. **1c**). On T2-weighted fat-suppressed axial imaging, the signal was slightly higher than that of skeletal muscle (Fig. **1d**). Diffusion-weighted imaging (DWI) with b = 800 s/mm^2^ displayed markedly increased signal intensity (Fig. **1e**). After enhancement, the mass showed a slight increase (Fig. **1f**).

#### 2.7.2 Case 2

Using a Samsung WS80A intracavitary probe for uterine and bilateral adnexal scanning, the left ovary shows a 2.3×2.3×1.7 cm cystic echo (Fig. **2a**) with 1.7×1.5×1.5 cm enhanced areas (Fig. **2b**), well-defined margins, and minimal blood flow signals, RI: 0.53 (Fig. **2c**). Pelvic MRI revealed an oval mass of approximately 2.3×2.1×2.0 cm in the left ovary. On T1-weighted axial imaging, the mass appeared isointense to skeletal muscle (Fig. **2d**). On T2-weighted fat-suppressed axial imaging, the signal was slightly higher than that of skeletal muscle (Fig. **2e**). DWI with b = 800 s/mm^2^ displayed markedly increased signal intensity (Fig. **2f**). After enhancement, no significant enhancement was observed in the mass (Fig. **2g**).

#### 2.7.3 Case 3

During a uterine and bilateral adnexal scan using a GE E6 intracavitary probe, a cystic mass measuring 5.1×3.9×3.2 cm with septa was identified on the surface of the right ovary (Fig. **3a**). The presence of slightly abundant blood flow above the septa (Fig. **3a**) suggests a potentially active lesion. The cystic mass contained fluid that was heterogeneous, with areas of clarity and viscosity, which is typical for ovarian cysts. Given the size of the cyst and the observed blood flow, further medical evaluation and monitoring are warranted. Pelvic MRI revealed a cystic mass in the posterior right aspect of the uterus, with relatively well-defined margins and dimensions of approximately 5.3×3.5×4.1 cm. On T1-weighted axial imaging, the mass presented with isointense signal intensity and contained multiloculated septations (Fig. **3c**). On T2-weighted fat-suppressed axial imaging, the mass presented with a mixed signal (Fig. **3d**). DWI with b = 800 s/mm^2^ displayed local high signal intensity (Fig. **3e**). The enhancement of the scan edges showed progressive intensification (Fig. **3f**).

#### 2.7.4 Case 4

Utilizing a GE E8 intracavitary probe to conduct scans of the uterus and bilateral adnexa, a 1.3×1.2 cm anechoic lesion was detected adjacent to the left ovary (Fig. **4**). Preoperatively, this lesion was misdiagnosed as a simple ovarian cyst.

The specimens underwent comprehensive harvesting and were subjected to HE staining, followed by sequential immunohistochemical staining. The tumor cells are arranged in a mixed structure of tubular (hollow or solid), cystic, reticular, or diffuse solid forms, partially located in a background of hyaline degenerated fibrous stroma. The lumen and reticular spaces of the nuclei often contain eosinophilic secretions. The tumor cells are cuboidal to oval, with sparse cytoplasm, warm and atypical nuclei, indistinct nucleoli, and rare mitotic (Fig. **5**). Immunohistochemical staining: CK (Fig. **6a**), inhibin (Fig. **6b**), WT-1 (Fig. **6c**), and calretinin (Fig. **6d**) were moderately to strongly positive in all 4 patients, GATA-3 (Fig. **6e**) and PAX 8 (Fig. **6f**) were all negative.

## 3 DISCUSSION

Female adnexal tumor of probable Wolffian origin (FATWO) is an extremely rare neoplasm originating from mesonephric (Wolffian) duct remnants. The majority of lesions develop in the broad ligament or mesosalpinx, while ovarian involvement is infrequent [1, 2]. Since its initial description in 1973, fewer than 100 cases have been reported globally [1]. Owing to the lack of specific clinical manifestations and imaging features, preoperative diagnosis remains arduous, and misdiagnosis is prevalent [2]. Definitive diagnosis depends on postoperative pathological examination. This study summarizes the ultrasound features of four pathologically confirmed FATWO cases and reviews the literature to enhance preoperative recognition. All four patients had no recurrence during follow-up (16–83 months), supporting the generally benign behaviour of FATWO [4, 5].

In our series, all four tumours were extraovarian (Figs. **1**-**4**), consistent with previous reports. Most lesions were well-circumscribed, solid or cystic-solid hypoechoic masses with mild to moderate internal vascularity. These features correspond directly to the ultrasound images (Figs. **1**-**4**). Our findings confirm previous ultrasonic features of FATWO: extraovarian origin, clear boundary, absent calcification, and mild internal vascularity [2, 6, 7].

The ultrasound features must be distinguished from sex cord-stromal tumours, fallopian tube tumours, and subserosal leiomyomas. Accurate recognition of extraovarian location (Figs. **1**-**4**) is the most important clue [8, 9].

FATWO has a low malignant potential. The reported age range is 15–81 years, with an average onset of approximately 50 years. Tumour size varies significantly, ranging from 0.8 cm to 20 cm [2, 5]. Most patients are asymptomatic, and lesions are often incidentally discovered during routine gynecologic examinations or surgeries. Although most FATWOs are benign, malignant cases have been reported. Diagnostic criteria for malignant FATWO have been established, including tumour diameter >10 cm, hypercellularity, necrosis, capsular invasion, rupture, tumour implantation, or metastasis [4]. Approximately 10% of patients experience recurrence or metastasis, most commonly in the liver and lungs, typically within 2 years [4, 5, 10].

Pathological diagnostic criteria for FATWO include [1, 2, 8]:

- Tumours originate from mesonephric duct remnants, most commonly in the broad ligament, mesosalpinx, or ovarian hilum.
- They are usually unilateral, with diameters ranging from 0.8 cm to 20 cm. The cut-surface is pale yellow or greyish-white, predominantly solid, but may be cystic or mixed cystic-solid.
- Histologically, tumours exhibit mixed architectural patterns, including solid, tubular, reticular, trabecular, and microcystic structures. Most cases present diffuse solid areas mixed with densely packed tubules. Luminal epithelial cells are cuboidal or low columnar, with scant cytoplasm, round or oval nuclei, and no significant atypia [1, 6].

There is no single specific immunohistochemical marker for FATWO. Previous studies have demonstrated consistent expression of CK, vimentin, CD10, calretinin, and inhibin, and the absence of PAX8 and GATA-3 [6, 11]. The four cases in this study displayed typical morphological features and immunohistochemical profiles consistent with previous reports, with no marked atypia, increased mitoses, or malignant features.

However, reports describing the imaging features of FATWO are still limited. By 2026, at least 18 cases with detailed MRI or CT findings have been documented in the English literature [4, 12-14].

As the first-line imaging modality for pelvic masses, ultrasound is crucial for the preoperative recognition of FATWO, yet dedicated ultrasound reports are extremely scarce. To clarify the contribution of the present study, we systematically compare our four cases with previously published ultrasound findings of FATWO [2, 8, 11].

### 3.1 Overlapping features consistent with previous ultrasound reports

Lesions are predominantly extraovarian, arising from the mesosalpinx or broad ligament, with identifiable normal ovarian tissue [1, 2, 8].

Masses are well-circumscribed with distinct borders [2, 8, 11].

Most lesions show internal vascularity with a resistive index (RI) ranging from 0.5 to 0.7 [1, 2].

The absence of calcification, papillary projections, and significant ascites is typical [1, 2, 8].

### 3.2 Confirmatory Findings Validated in the Present Study

A solid hypoechoic appearance is the most common pattern [2, 8].

Cystic-solid components and internal septations may be present [11].

Tumors are typically unilateral and encapsulated [1, 2, 6].

### 3.3 Novel Findings of this Study (Not Previously Emphasized)

We clarify that capsular vascularity and intratumoral vascularity can coexist, which aids in distinguishing FATWO from leiomyoma.

We provide a standardized ultrasound diagnostic approach based on a four-case cohort, including precise localization, echo characterization, and vascular analysis.

By distinguishing overlapping, confirmatory, and novel ultrasonic features, this study strengthens the ultrasonographic diagnostic criteria for FATWO. The present series represents one of the largest ultrasound cohorts of FATWO and provides more reliable features for preoperative clinical suspicion.

Since FATWO arises from mesonephric duct remnants, it occurs along the migratory path of the Wolffian duct, including the ovarian hilum, mesosalpinx, lateral uterus, and upper vagina. Most lesions are extraovarian, and careful scanning often identifies a normal ovary. Accurate localization is thus beneficial for diagnosis [1, 2, 6].

The sonographic appearance must be differentiated from:

#### 3.3.1 Sex cord-stromal Tumors

Cystic-solid lesions such as granulosa cell tumors may resemble FATWO, but these almost never occur outside the ovary [6, 9].

#### 3.3.2 Fallopian Tube Tumors

Hydrosalpinx appears tubular or sausage-shaped; malignant tubal tumors show irregular masses, papillary projections, and are often associated with effusion [1, 2].

#### 3.3.3 Subserosal or Broad Ligament Leiomyomas

These may exhibit degenerative cystic changes, but their anatomical relationship to the uterus and vascular characteristics differ from FATWO [1, 2].

When a well-defined hypoechoic or mixed adnexal mass is detected, especially with an extraovarian location, FATWO should be included in the differential diagnosis. MRI and CT may offer complementary information, but the gold standard remains surgical pathological diagnosis [4, 8, 12-14].

Recent studies using next-generation sequencing have shown that FATWO lacks common mutations such as KRAS/NRAS, PTEN, PIK3CA, or CTNNB1 [4]. A subset of cases shows STK11 alterations [13]. Most tumors behave benignly, and treatment involves complete surgical resection with long-term follow-up. The role of chemotherapy, radiotherapy, or hormonal therapy for malignant cases remains unclear [3, 5]. All four patients in this study were alive with no evidence of disease (NED) at the last follow-up (range: 16–83 months).

## CONCLUSION

Given the nonspecific imaging features of FATWO, a preoperative diagnosis remains challenging. The present cases suggest that a well-defined extra-ovarian solid or cystic-solid adnexal mass with mild internal blood flow could raise suspicion for FATWO and warrant inclusion in the differential diagnosis.

FATWO should be included in the differential diagnosis of well-circumscribed extraovarian solid or cystic-solid adnexal masses detected by ultrasound, even when the appearance mimics common benign ovarian lesions.

## AUTHOR’S CONTRIBUTIONS

The authors confirm contribution to the paper as follows: L. X., K.C., J.W.: Study conception and design; L.Y., L.X.: Data collection; K.C., L.Y., L.X.: Analysis and interpretation of results; L.Y., L.X., J.W.: Draft manuscript. All authors reviewed the results and approved the final version of the manuscript.

## Figures and Tables

**Fig. (1):**
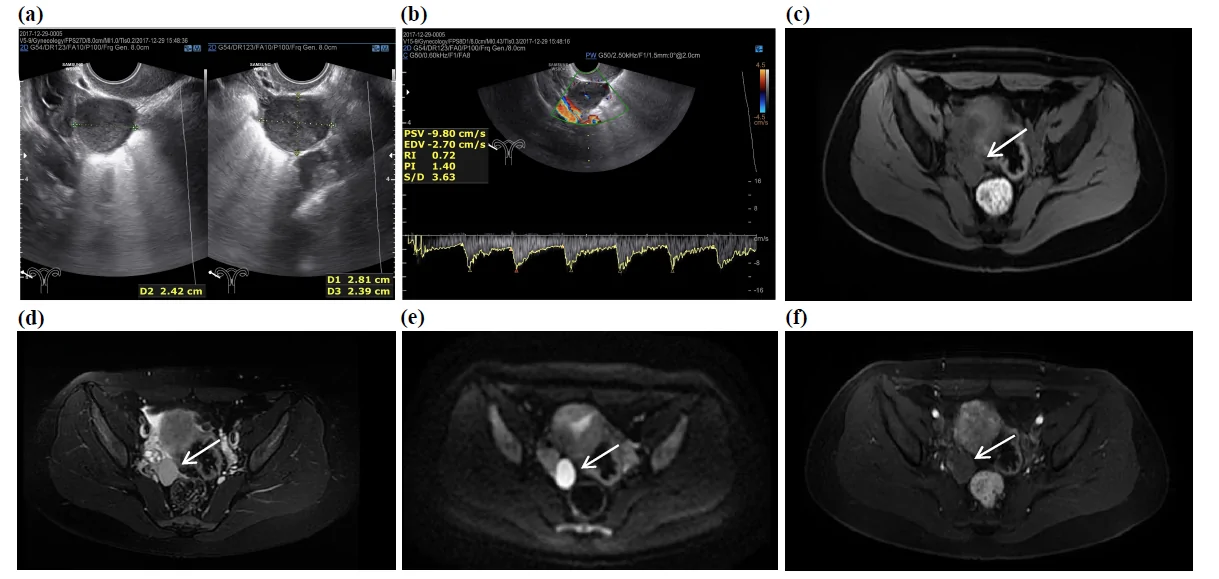
Transvaginal ultrasound and pelvic MRI findings of Case 1. (**a**) Transvaginal ultrasound shows a well-defined hypoechoic mass adjacent to the right ovary. (**b**) Color Doppler imaging demonstrates internal blood flow within the mass. (**c**) Axial T1-weighted MRI shows the mass with isointense signal relative to skeletal muscle. (**d**) Axial fat-saturated T2-weighted MRI shows slightly hyperintense signal of the mass. (**e**) Diffusion-weighted imaging (b = 800 s/mm^2^) shows marked hyperintensity of the lesion. (**f**) Contrast-enhanced T1-weighted MRI reveals mild enhancement of the mass.

**Fig. (2):**
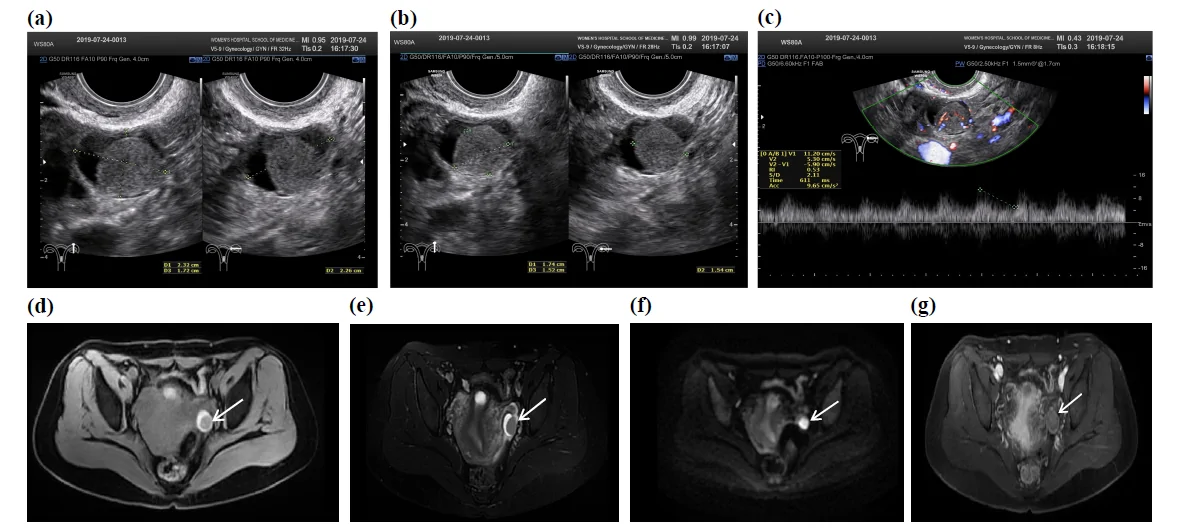
Transvaginal ultrasound and pelvic MRI findings of Case 2. (**a**) Transvaginal ultrasound shows a cystic lesion in the left adnexal region. (**b**) Gray-scale ultrasound identifies a solid enhancing component within the cyst. (**c**) Color Doppler imaging shows minimal blood flow signals in the lesion. (**d**) Axial T1-weighted MRI shows the mass with isointense signal relative to muscle. (**e**) Axial fat-saturated T2-weighted MRI shows a slightly hyperintense signal of the mass. (**f**) Diffusion-weighted imaging (b = 800 s/mm^2^) demonstrates marked hyperintensity. (**g**) Contrast-enhanced T1-weighted MRI shows no significant enhancement.

**Fig. (3):**
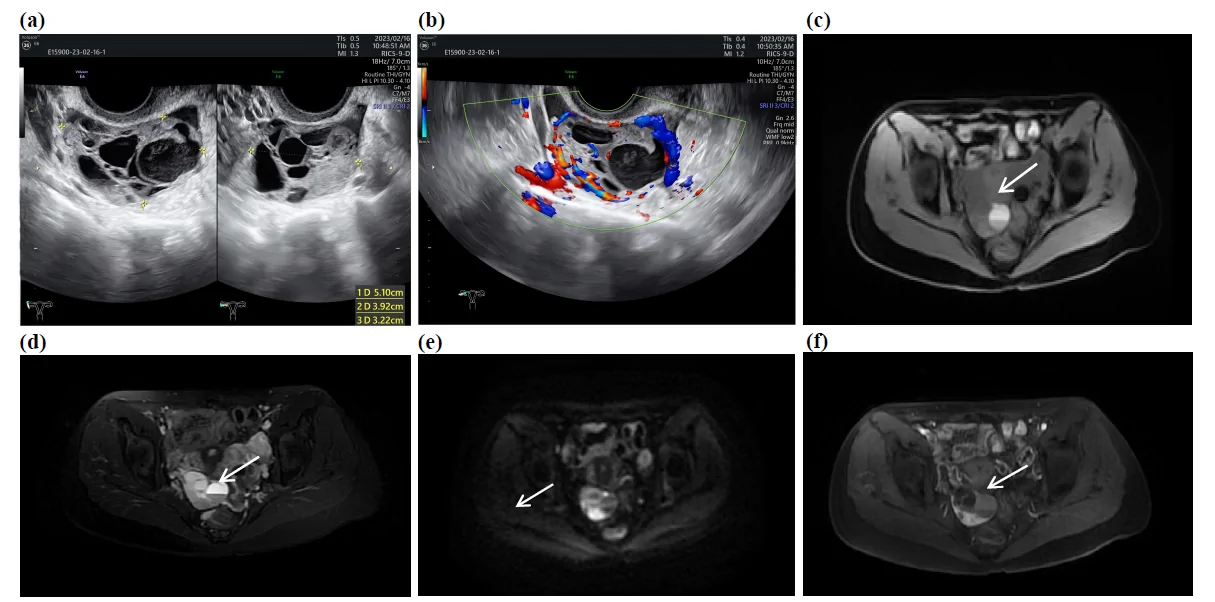
Transvaginal ultrasound and pelvic MRI findings of Case 3. (**a**) Transvaginal ultrasound reveals a septated cystic mass on the right ovarian surface. (**b**) Color Doppler imaging shows increased blood flow along the internal septations. (**c**) Axial T1-weighted MRI shows a multiloculated cystic mass with isointense septa. (**d**) Axial fat-saturated T2-weighted MRI shows mixed signal intensity within the mass. (**e**) Diffusion-weighted imaging (b = 800 s/mm^2^) shows focal hyperintensity. (**f**) Contrast-enhanced T1-weighted MRI demonstrates progressive marginal enhancement.

**Fig. (4):**
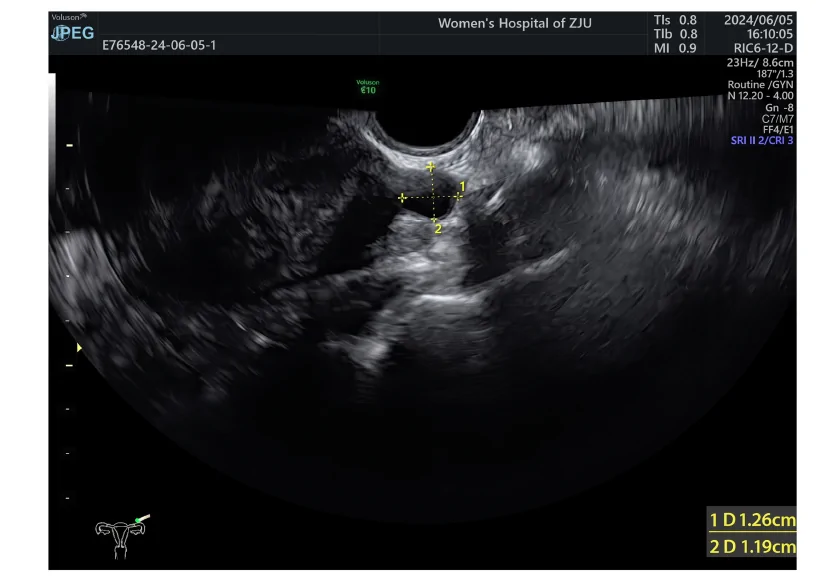
Transvaginal ultrasound finding of Case 4. Transvaginal ultrasound shows a small anechoic lesion adjacent to the left ovary, misdiagnosed preoperatively as a simple ovarian cyst.

**Fig. (5):**
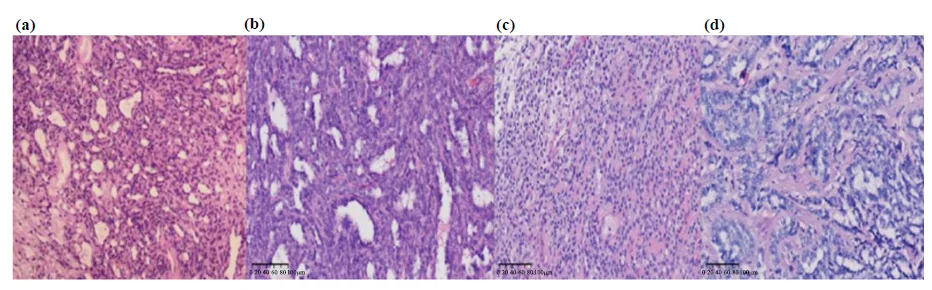
Histopathological features of FATWO (HE staining, ×200). (**a**-**d**) Tumor cells exhibit mixed tubular, cystic, reticular and solid architectures, embedded in a hyalinized fibrous stroma.

**Fig. (6):**
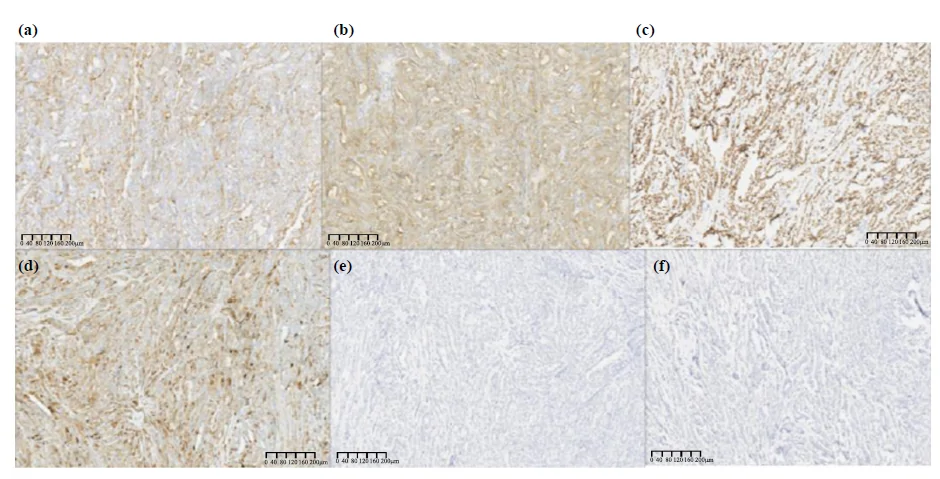
Immunohistochemical staining of FATWO (EnVision method, ×100). (**a**) CK positivity; (**b**) inhibin positivity; (**c**) WT-1 positivity; (**d**) calretinin positivity; (**e**) GATA-3 negativity; (**f**) PAX8 negativity.

**Table 1:** Summary of 4 cases of tumors possibly originating from wolffian duct appendages.

-	**Case 1**	**Case 2**	**Case 3**	**Case 4**
Age	27 years old	42 years old	41 years old	53 years old
Action in chief	Pelvic mass detected during physical examination	Discovery of pelvic mass 4 months	Discovery of pelvic mass and cervical abnormalities 8 months ago	Menstrual disorders for 2 months
Laboratory examination	Negative	Negative	Negative	Negative
Ultrasound location	Next to the right ovary	Within the left ovary (actually the left mesosalpinx)	Surface of the right ovary	Adjacent to the left ovary, broad ligament
Ultrasound size	2.7×2.0×2.5 cm	2.3×2.3×1.7 cm	5.1×3.9×3.2 cm	1.3×1.2 cm
Ultrasonic echo	Solid hypoechoic	Solid mass with slightly hyperechoic area within	Cystic predominance with septation	Small cystic echo with good internal acoustic transmission
Blood flow RI	Visible blood flow, RI 0.72	Minimal blood flow, RI 0.53	The septum exhibits slightly increased blood flow	No significant blood flow was observed.
Preoperative imaging diagnosis	Solid nodule adjacent to the right ovary (fibroma?)	Cystic mass in the left ovary (luteal cyst/endometriotic cyst?)	Cystic tumor of the right ovary (granulosa cell tumor?)	Dark area adjacent to the left ovary
Intraoperative position	Right fallopian tube mesentery	Mesentery of the ampulla of the left fallopian tube	Mesentery of the fimbriated end of the right fallopian tube	Left lateral ligament, not connected to the uterine body
Intraoperative size	Approximately 3×3×2 cm	Approximately 3 cm	The diameter is approximately 5 cm.	The diameter is approximately 2.0 cm.
Intraoperative gross anatomy	Pedunculated, smooth, soft in texture, with clear margins	Solid and cystic, with smooth surface	Uneven wall thickness, internal septation, partially solid, yellowish and brittle	Nodules, penetrating the anterior leaf of the broad ligament
Pathological structure	A mixture of tubular, saccular and reticular forms	Mixed tubular, cystic, and reticular patterns	Mixed tubular, cystic, and reticular patterns	Mixed tubular, cystic, and reticular patterns
Immunohistochemical	CK, inhibin, WT-1, calretinin positive;GATA-3, PAX8 negative	CK, inhibin, WT-1, calretinin positive;GATA-3, PAX8 negative	CK, inhibin, WT-1, calretinin positive;GATA-3, PAX8 negative	CK, inhibin, WT-1, calretinin positive;GATA-3, PAX8 negative
Follow-up	83 months, NED	75 months, NED	32 months, NED	16 months, NED

## Data Availability

The data used to support the findings of this study are available from the corresponding author [J.W] upon request.

## LIST OF ABBREVIATIONS

CARE: Case Report Guidelines
CK: Cytokeratin
DWI: Diffusion-Weighted Imaging
FATWO: Female Adnexal Tumor of Probable Wolffian Origin
GATA-3: GATA Binding Protein 3
HE: Hematoxylin and Eosin
MRI: Magnetic Resonance Imaging
NED: No Evidence of Disease
PAX8: Paired Box 8
RI: Resistive Index
WT-1: Wilms Tumor Protein 1

## STANDARDS FOR REPORTING

CARE guidelines and methodology were followed.
